# Efficacy and Cerebrospinal Fluid Rhinorrhea after Cabergoline Treatment in Patients with Bioactive Macroprolactinoma

**DOI:** 10.3390/cancers13215374

**Published:** 2021-10-26

**Authors:** Hae-Kyung Kim, Jae-Won Hong, Ju-Hyung Moon, Sung-Soo Ahn, Eui-Hyun Kim, Seung-Koo Lee, Eun-Jig Lee, Yae-Won Park, Cheol-Ryong Ku

**Affiliations:** 1 Department of Internal Medicine, Institute of Endocrine Research, Yonsei University College of Medicine, Seoul 03722, Korea; hkkim1@yuhs.ac (H.-K.K.); EJLEE423@yuhs.ac (E.-J.L.); 2 Pituitary Tumor Center, Severance Hospital, Seoul 03722, Korea; MJJR80@yuhs.ac (J.-H.M.); SUNGSOO@yuhs.ac (S.-S.A.); EUIHYUNKIM@yuhs.ac (E.-H.K.); SLEE@yuhs.ac (S.-K.L.); 3 Department of Internal Medicine, Division of Endocrinology, Ilsan-Paik Hospital, Inje University College of Medicine, 170 Juhawro, Ilsanseo-gu, Goyang 10380, Korea; hjwcarrot@paik.ac.kr; 4 Department of Neurosurgery, Yonsei University College of Medicine, Seoul 03722, Korea; 5 Center for Clinical Imaging Data Science, Department of Radiology, Research Institute of Radiological Science, Yonsei University College of Medicine, Seoul 03722, Korea

**Keywords:** cabergoline, drug resistance, cerebrospinal fluid rhinorrhea, prolactinoma

## Abstract

**Simple Summary:**

Predicting dopamine agonist resistance in patients with macroprolactinoma is essential for clinicians to prevent treatment failure and subsequent complications such as medication–induced cerebrospinal fluid (CSF) rhinorrhea. In this retrospective cross-sectional study that included 140 patients with newly diagnosed and cabergoline-treated prolactinoma with prolactin levels ≥1000 ng/mL, non-responders and patients with CSF rhinorrhea included a significantly higher number of patients receiving hormone replacement therapy than responders. Hormone deficiency was associated with a greater odds ratio for the risk of non-responders (adjusted odds ratio = 5.13, 95% CI 1.96–13.46, *p* = 0.001). Cabergoline was effective in bioactive macroprolactinoma and initial cabergoline dose in long-term responsiveness and development of CSF rhinorrhea was not significantly different. Hypopituitarism was independently associated with an increased risk of cabergoline resistance and CSF rhinorrhea.

**Abstract:**

Predicting dopamine agonist resistance in patients with macroprolactinoma is essential for clinicians to prevent treatment failure and subsequent complications such as medication-induced cerebrospinal fluid (CSF) rhinorrhea. We evaluated the features of patients with cabergoline resistance and CSF rhinorrhea in patients with prolactinomas with prolactin levels ≥1000 ng/mL. A total of 140 patients who were newly diagnosed with prolactinoma secreting only prolactin ≥1000 ng/mL and treated with cabergoline for the first time were included in this study. Based on the hormonal and radiologic response of the prolactinoma, the patients were divided into responders and non-responders. Non-responders (36/140, 25.8%) included a higher number of patients receiving hormone replacement than responders (responders, *n* (%) = 12(11.5) vs. non-responders = 13(36.1), *p* = 0.001). In propensity score matching analysis, patients who developed CSF rhinorrhea presented more frequent hormone deficiency than responders regardless of initial cabergoline dose. Hormone deficiency was associated with a greater odds ratio for the risk of non-responders (adjusted odds ratio = 5.13, 95% CI 1.96–13.46, *p* = 0.001). Cabergoline was effective in bioactive macroprolactinoma. Furthermore, initial cabergoline dose was not significantly associated with long-term responsiveness and development of CSF rhinorrhea but the hypopituitarism was independently associated with an increased risk of cabergoline resistance and CSF rhinorrhea.

## 1. Introduction 

Giant prolactinomas are defined as adenomas with tumor diameters ≥ 4 cm and prolactin levels (PRLs) ≥ 1000 ng/mL, accounting for only 0.5% of all pituitary adenomas and are predominantly found in men [1,2,3]. The treatment goals for giant prolactinomas include normalization of PRL with recovery of altered pituitary axes, but, more importantly, reduction of tumor size with relief of compression of adjacent structures, particularly the optic chiasm and cranial nerves [4].

Currently, cabergoline, a dopamine agonist (DA), is generally the first-line treatment of choice for prolactinomas [5], which reduces the size of tumor and normalizes PRL, leading to alleviation of endocrine and neurologic symptoms in 80–95% of patients during the first month of treatment [6,7]. Surgical approach is another choice for patients intolerant or resistant to DA and for those who develop acute complications such as cerebrospinal fluid (CSF) rhinorrhea, apoplexy, or chiasmal herniation [4]. DA-induced CSF rhinorrhea is a detrimental medical complication caused by DA resistance that occurs during the first 3 months and has mortality rate of 25–50%, requiring emergent surgical intervention [8,9,10]. However, surgical resection of prolactinoma pretreated with DA demonstrated a low remission rate due to fibrotic changes of the tumor [11]. Therefore, identifying patients with a high risk of DA resistance is pivotal in order to avoid second-line surgery and subsequent complications like CSF rhinorrhea.

Previous studies identified the clinical characteristics of DA resistance such as young age; male; macroadenoma; cavernous sinus invasion; cystic, hemorrhagic, and necrotic transformations of tumors; and genetic predisposition [12,13]. However, due to inaccessibility of tissue confirmation, studies exclusively involving bioactive macroprolactinoma, in which the first-line treatment between medical or surgical approach should be distinguished to achieve high remission rates, are currently lacking. 

In this study, we evaluated the long-term responses of cabergoline in patients with bioactive macroprolactinoma with PRL ≥ 1000 ng/mL and examined the clinical and radiologic features of patients with cabergoline resistance and cabergoline-induced CSF rhinorrhea.

## 2. Results 

### 2.1. Baseline Characteristics of the Study Population Based on the Hormonal and Radiologic Response to Cabergoline

A total of 140 patients (108 men and 32 women) with a mean age of 43 years were enrolled in this study. The patients were divided into responders (*n* = 104) and non-responders (*n* = 36) based on their hormonal and radiologic response to cabergoline (Table 1). Non-responders were characterized by a higher prevalence of hypopituitarism requiring hormone replacement therapy than responders but no significant difference in hormone deficiency recovery. The basal PRL before the initiation of cabergoline was 2647 ng/mL in responders and 2892 ng/mL in non-responders (*p* = 0.34) and the median time to normalization of PRL was 3 months (Interquartile range, IQR 3–15 months). Age, first month of cabergoline dose, total cabergoline dose in the first year, and duration of cabergoline treatment showed no difference between the two groups. No significant difference was observed in the basal FSH and testosterone levels between responders and non-responders.

The radiologic characteristics were analyzed between responders and non-responders. Before cabergoline treatment, the most dominant Knosp grade was grade IV (*n* = 85, 60.7%), but there was no significant difference between the two groups even after being dichotomized by grade IIIB and above. The cystic and hemorrhagic components in tumors were similar, and the majority of tumors invaded the cavernous sinus (*n* = 115, 82.1%) and skull base (*n* = 137, 97.9%), with no significant difference between the two. Sphenoid sinus involvement was predominantly post-sellar (*n* = 84, 60%), and sphenoid sinus pneumatization showed no difference between the two groups. The T2 SI ratio at baseline was similar between the two groups. The median tumor volume was 6.7 cm^3^, and the maximal tumor diameter was 29 mm. The tumor volume, prolactin index, and maximal tumor diameter showed no significance between the two groups.

### 2.2. Baseline Clinical Characteristics of the Study Population Based on the Hormonal or Radiologic Response to Cabergoline

The patients were divided into biochemical responders and biochemical non-responders by normalization of PRL (Table 2). As in the previous results of the baseline clinical characteristics of the study population based on both hormonal and radiologic responses (Table 1), biochemical non-responders included a larger number of patients who received steroid replacement therapy but not thyroid or sex hormone replacement therapy. Age, first month of cabergoline dose, total cabergoline dose in the first year, and duration of cabergoline treatment showed no difference between the two groups. The basal PRL was 2620 ng/mL in biochemical responders and 3827 ng/mL in biochemical non-responders (*p* = 0.21). The basal FSH level showed no significant difference between the two groups, but the basal testosterone level was lower in biochemical non-responders (136 vs. 72 ng/dL, *p* = 0.013). Among biochemical responders, 92.9% (104/112) were biochemical and radiologic responders. Other radiologic parameters were not significantly different. The baseline characteristics of patients divided by radiologic responses to cabergoline are described in Table S1. Among radiologic responders, 83.1% (103/124) were biochemical and radiologic responders. 

### 2.3. Clinical Characteristics of Patients with CSF Rhinorrhea

PSM was performed among hormonal and radiologic responders without CSF rhinorrhea and patients with CSF rhinorrhea, and 14 responders were selected after adjusting for age, sex, and tumor volume (Table 3). Seven patients had CSF rhinorrhea and the median period between the initiation of cabergoline and CSF rhinorrhea was 1 month (IQR 1–2 months). A majority of patients 71.4% (5/7) experienced CSF rhinorrhea within 1 month, and another two patients experienced it within 2 and 3 months. The majority of patients with CSF rhinorrhea were males (*n* = 6, 85.7%) with a mean age of 39.9 years. The median cabergoline dose in the first month in both groups was 2 mg/week. None of the responders required hormone replacement; therefore, there were significant differences in steroid, thyroid, and sex hormone replacement between the two groups. Since patients with CSF rhinorrhea underwent transsphenoidal surgery, their total cabergoline dose in the first year was lower than that of responders. The difference of basal radiologic features was not significantly different between patients with CSF rhinorrhea and hormonal and radiologic responders. 

Patients with CSF rhinorrhea were compared with hormonal and radiologic non-responders who were selected by the PSM method previously applied to choose responders (Table S2). The number of patients who received hormone replacement therapy was similar between the two groups, but patients with CSF rhinorrhea required more steroid replacement than non-responders. All patients with CSF rhinorrhea demonstrated radiologic features such as Knosp grade IV, cavernous sinus, skull base, sphenoid sinus post-sellar involvement, and sphenoid sinus pneumatization, which were not significantly different from those of non-responders. The T2 SI ratio, prolactin index, tumor volume, and maximal diameter were not significantly different between patients with CSF rhinorrhea and non-responders. 

### 2.4. Risk of Cabergoline Resistance in Non-Responder in Bioactive Macroprolactinoma 

The risk of cabergoline resistance in hormonal and radiologic non-responders in relation to potential confounders was analyzed using logistic regression analyses (Table 4). Hormone replacement therapy was significantly associated with a risk of cabergoline resistance in hormonal and radiologic non-responders, but basal PRL and radiologic features did not exhibit a significant risk in relation to non-responders. Even after adjusting multiple confounding parameters, hormone deficiency was associated with a greater odds ratio for the risk of resistance in non-responders (Aor = 5.13, 95% CI 1.96–13.46, *p* = 0.001, Table 5). 

## 3. Discussion 

Identifying risk factors of DA resistance is important for clinicians since there is no current consensus or guideline to predict DA resistance in prolactinoma or serious complications such as CSF rhinorrhea. Since PRL ≥ 1000 ng/mL was considered an important feature for creating a milieu of DA resistance in previous studies [14,15], we adopted the term bioactive prolactinoma to analyze a broader spectrum of DA-resistant prolactinoma. To our knowledge, this study involved the largest study population of 140 patients with newly diagnosed and cabergoline-treated bioactive prolactinoma with a PRL ≥ 1000 ng/mL, regardless of tumor size, and demonstrated that hypopituitarism is significantly associated with cabergoline non-responsiveness and cabergoline-induced CSF rhinorrhea. 

The DA resistance rate was 25.8% in patients with bioactive macroprolactinoma in the present study, which was 10% to 18% higher than that of patients with prolactinoma in general [11] but similar to those with macroprolactinoma (29%) [9], which corresponds with our hypothesis that bioactive prolactinoma with PRL ≥ 1000 ng/mL creates an environment of DA resistance unlike general prolactinoma. Furthermore, bioactive prolactinoma exhibited clinical and radiologic features resembling giant prolactinoma, such as predominance in male sex, median age of 42 years, hypopituitarism, invasion of cavernous sinus, and post-sellar extension into sphenoid sinus [16]. Generally, symptoms such as hypogonadism, visual defects, and headaches in men are typically overlooked for a longer period of time compared with those in women, which is why prolactinomas in men are in more advanced stages when they are first diagnosed [17], developing more complications such as hypopituitarism and subsequent post-treatment complications such as CSF rhinorrhea. There was a significant difference in hormone replacement therapy between patients with CSF rhinorrhea and responders or non-responders. Even after adjusting multiple variables associated with DA resistance, hypopituitarism was independently associated with an increased risk of resistance in non-responders with bioactive prolactinoma.

DA reduces the secretion of prolactin by binding to the dopamine 2 receptor (D2R) in the pituitary tumor, consequently decreasing the tumor volume and lowering the angiogenesis in the surrounding tissue and inducing a tumoricidal effect [18]. The main pathogenesis of DA-resistant prolactinomas includes decreased expression of D2R, resulting in the biologic transformation of tumors [19,20]. In this study, we attempted to conjugate previously discovered DA resistance-associated risk factors in patients with prolactinoma with a high PRL to discover significant features related to non-responders and patients with CSF rhinorrhea. In concordance with previous studies, age, male predominance, size of tumor, and cavernous sinus invasion were found in patients with bioactive prolactinoma in this study. Moreover, we discovered that hypopituitarism was significantly associated with non-responders in bioactive prolactinoma. 

Sequential anterior hypopituitarism due to pituitary tumor enlargement is well established [21,22]. Predominant pathophysiology of hypopituitarism caused by pituitary adenomas involves mechanical compression of portal vessels and pituitary stalk and ischemic necrosis of anterior lobe [23]. Moreover, an increase in intrasellar pressure in patients with macroadenomas not only causes reduced blood flow through the portal vessels and the pituitary stalk, but also diminished delivery of hypothalamic hormones to the anterior pituitary [24]. The hormone deficiency usually improves after normalization of PRL and reduction of tumor size in patients with macroprolactinomas [16]. However, reversibility of pituitary dysfunction is controversial and a prospective study of 12 patients with macroprolactinoma treated with cabergoline showed that pituitary dysfunction, except for gonadotroph axis, was mostly irreversible even after achieving normoprolactinemia [25]. In this study, non-responders and patients with CSF rhinorrhea received more hormone replacement therapy at diagnosis and showed a relatively lower rate of recovery of hormone deficiency than responders (responders *n* = 5/12 (41.7%) vs. *n* = 2/13(15.4%)), implying that a prolonged mass and stalk compression effect of prolactinoma with high bioactivity subsequently induces intradenomatous apoplexy and destruction of anterior pituitary cells, leading to an irreversible anterior pituitary hormone deficiency. In summary, an advanced stage of prolactinoma with a high PRL and hormone deficiency may represent biologic transformation of prolactinoma with destroyed pituitary cells, decreasing the chances for DA to bind to D2R in pituitary cells and resulting in DA resistance. Further investigations with prospective design for detailed mechanisms of the association between DA resistance in bioactive prolactinoma and hypopituitarism are warranted in the future. 

Previous studies have reported that a rapid dose escalation of cabergoline treatment in patients with macroprolactinoma may lead to massive shrinkage with the potential risk of apoplexy or CSF rhinorrhea, with incidence rate 6.1% [8,26,27]. In this study, CSF rhinorrhea occurred within the first 3 months of cabergoline in concordance with a previous study [28] but the initial dose of cabergoline was not significantly different between patients with CSF rhinorrhea and responders and non-responders by PSM. Patients with CSF rhinorrhea received more hormone replacement compared with responders or non-responders. As described earlier, hormone deficiency may represent a prolonged advanced status of prolactinoma with intra-adenomatous apoplexy, which might cause CSF rhinorrhea regardless of the initial dose of cabergoline. In summary, clinicians should be aware of treatment choices between medical or surgical approach in patients with prolactinoma with PRL ≥ 1000 ng/mL and hormone deficiency, which may reflect DA resistance and a higher risk of CSF rhinorrhea. 

This study has several limitations. First, a causal relationship between hypopituitarism and non-responders could not be determined due to the cross-sectional design of the study. Larger prospective studies are needed to validate the relationship between hypopituitarism and DA resistance in the future. Second, this study did not confine the tumor size to 4 cm, which is the standard diameter of giant prolactinoma. In clinical practice, deciding the treatment option does not particularly depend on whether tumor size exceeds 4 cm or not. Therefore, patients with overt bioactive prolactinomas with PRL ≥ 1000 ng/mL were included to incorporate patients who might require second-line surgery, which could provide clinicians with hints for a further treatment plan after the initiation of cabergoline treatment. Third, visual acuity examination or combined pituitary function test was not performed in all patients. Therefore, relief of clinical symptoms by mass effect of macroadenoma was not fully examined, which could be one of the parameters to predict cabergoline resistance and CSF rhinorrhea. Additionally, recovery of hormone function was not based on the recovery of symptoms of hypogonadism or combined pituitary function test; the percentage of hormone deficiency recovery could be underestimated. Lastly, due to a recent guideline of diagnosing hypopituitarism and risk of growth hormone replacement in macroadenoma, the diagnosis of hypopituitarism including growth hormone deficiency was not detected through combined pituitary function test. 

## 4. Materials and Methods 

### 4.1. Study Design and Population 

Between November 2005 and June 2020, 140 patients from a single tertiary hospital were included in this retrospective study. We enrolled patients aged ≥ 19 years with the following inclusion criteria: (1) clinically and biochemically confirmed bioactive prolactinoma with serum PRL ≥ 1000 ng/mL and symptoms of galactorrhea, oligomenorrhea/amenorrhea, or male hypogonadism; (2) patients who underwent a sellar magnetic resonance imaging (MRI) scan at baseline and after cabergoline treatment; and (3) cabergoline prescription. The exclusion criteria were as follows: (1) co-secreting tumor, (2) previous history of prolactinoma treatment (medication, surgery, or radiation), and (3) cancer. Responders were defined as patients with normalization of PRL (20 ng/mL for males and 25 ng/mL for females) and a ≥50% tumor volume reduction (TVR) at the last follow-up [6]. Non-responders were defined as patients with failed normalization of PRL and no response in terms of TVR < 50% after cabergoline treatment [6]. Biochemical responders and non-responders were defined dependent solely on normalization of PRL. 

Demographic data such as age, sex, and cabergoline dose in the first month, total dose of cabergoline in 1 year, duration of cabergoline use, and hormone replacement therapy were obtained. Hormone replacement therapy was defined as treatment with any of the following medications that the patients were prescribed at initial diagnosis of prolactinoma based on the basal pituitary hormone assay and combined pituitary function test: steroid, thyroid hormone, or sex hormone. The recovery of hormone deficiency was defined by complete discontinuation of hormone replacement therapy. The study protocol was reviewed and approved by the Institutional Review Board of Yonsei University College of Medicine (No. 4-2020-1274), and the need for informed consent was waived because of the retrospective nature of the study. 

### 4.2. Treatment and Follow-Up Protocol 

All patients were started on cabergoline treatment in a pituitary center by experienced endocrinologists specializing in the field of pituitary diseases [29,30,31]. Cabergoline was administered at an initial dose of 0.5–4 mg weekly, depending on the size of the prolactinoma with dose adjustments depending on prolactin response and patient tolerance [32]. All patients underwent blood tests, basal pituitary hormone assay, and MRI scans of the sellar area before treatment. PRL was measured every 3 months for 1 year and 6 months thereafter, and follow-up MRI was performed at 15 and 27 months, annually or biennially. Surgical intervention was performed for patients with uncontrolled PRL and symptoms caused by mass effect despite cabergoline dose escalation. FSH levels in females and testosterone levels in males were measured before treatment and at follow-up. CSF rhinorrhea was confirmed in all cases during examination by a neurosurgeon, biochemical analysis of fluid for glucose, and computed tomography-cisternography [28]. When CSF rhinorrhea was detected, patients underwent reconstruction of the defect and tumor resection by transsphenoidal surgery. 

### 4.3. Imaging Assessment 

Radiologic features were assessed on sellar MRI by two neuroradiologists who were blinded to clinical follow-up and laboratory data. The radiologic features included Knosp grading [33], cystic and hemorrhagic components, cavernous sinus, skull base, and sphenoid sinus involvement and pneumatization [7,34]. T2 signal intensity (SI) ratios were measured, and tumor volume was calculated using the Di Chiro and Nelson formula: volume = height × length × width × π/6 [35]. Prolactin index was calculated by dividing serum PRL concentration by tumor volume [36].

### 4.4. Measurement of Serum Prolactin 

Serum PRLs were measured by Coat-A-Count prolactin immunoradiometric assay (Siemens; within-run coefficient of variation: 1.1–2.7%; run-to-run coefficient of variation: 1.6–6.3%; conversion factor: ng/mL × 21.2 = mIU/L) The reference range was 3.1–16.5 ng/mL for males and 3.6–18.9 ng/mL for females [37]. All samples of patients were diluted to overcome the hook effect to avoid false-negative results [1,38]. 

### 4.5. MRI Protocol 

Patients were scanned on various 1.5 or 3.0 Tesla MRI units (Achieva/Ingenia/Ingenia CX from Philips Medical Systems or Discovery MR750 from GE Healthcare, Chicago, IL, USA). The protocol included coronal T1-weighted turbo spin-echo images and coronal T2-weighted turbo spin-echo images. Postcontrast coronal T1-weighted turbo field echo images were acquired 2–4 min after 0.1 mmol/kg of Gadolinium contrast agent (Dotarem; Guerbert, Aulnay Sous Bois, France) was fully injected at a rate of 2 mL/s. 

### 4.6. Statistical Analysis 

The data are presented as mean ± standard deviation for normally distributed continuous variables and medians [interquartile range (IQR)] for non-normally distributed continuous variables. The baseline characteristics of the study population were analyzed using the two-sample Student’s *t*-test for continuous variables and the chi-square test for categorical variables. We used 1:2 propensity score matching (PSM) to compare patients with CSF rhinorrhea and non-responders or responders with propensity scores estimated using non-parsimonious multiple logistic regression model with variables such as age, sex, and tumor volume and the nearest neighbor matching algorithm to balance the covariates between the two groups, thereby reducing selection bias [39]. Univariate logistic regression analyses were used to determine the independent factors associated with the incidence of non-responders in bioactive prolactinoma, and multiple logistic regression analysis was performed to evaluate the independent association between significant factors deduced from the univariate analysis and non-responders, with adjustment for age, sex, total cabergoline dose in the first year, basal PRL, basal tumor volume, sphenoid sinus post-sellar involvement, and sphenoid sinus pneumatization. Statistical analyses were performed using the IBM SPSS statistical software for Windows, version 25.0 (IBM, Armonk, NY, USA). Adjusted odds ratios (aORs) and 95% confidence intervals (Cis) were determined, and *p* < 0.05 was considered statistically significant.

## 5. Conclusions

To our knowledge, this is the first study demonstrating that hypopituitarism is independently associated with an increased risk of DA resistance in patients treated with cabergoline in bioactive macroprolactinoma with PRL ≥ 1000 ng/mL. Furthermore, we discovered that patients with CSF rhinorrhea required more hormone replacement than non-responders or responders, regardless of age, sex, and tumor volume. The clinical implication of hypopituitarism in patients with prolactinoma to predict cabergoline resistance and CSF rhinorrhea needs to be validated in the future.

## Figures and Tables

**Table 1:** Baseline characteristics and radiologic characteristics of bioactive prolactinoma according to hormonal and radiologic response of cabergoline.

	Total (*n* = 140)	Responder (*n* = 104)	Non-Responder (*n* = 36)	*p* Value
Demographics				
Age (years)	43 ± 14	43 ± 14	43 ± 16	0.77
Male sex (*n*, %)	108(77.1)	80(76.9)	28(77.8)	0.92
First month CAB dose (mg/week)	2(1–2)	2.0(1.0–2.0)	2.0(1.3–3.0)	0.37
Hormone replacement therapy (*n*, %)	25(17.9)	12(11.5)	13(36.1)	**0.001**
Steroid replacement (*n*, %)	10(7.1)	4(3.8)	6(17.1)	**0.008**
Thyroid hormone replacement (*n*, %)	16(11.4)	8(7.7)	8(22.2)	**0.018**
Steroid and thyroid hormone replacement (*n*, %)	6(4.3)	2(1.9)	4(11.1)	**0.019**
Sex hormone replacement (*n*, %)	10(7.1)	4(3.8)	6(16.7)	**0.01**
Hormone deficiency recovery (*n*, %)	7(7.6)	5(4.8)	2(5.6)	0.14
Total CAB dose in 1st year (mg/year)	118(92–144)	100(92–144)	138(92–179)	0.57
Duration of CAB use (months)	65.5(31–90)	62(31–90)	73(28–91)	0.99
**Hormones (Basal)**				
Prolactin (ng/mL)	2592(1584–4775)	2647(1535–4728)	2892(1932–6049)	0.34
**Radiologic features (Basal)**				
Grade of Knosp				
Grade 0 (*n*, %)	5(3.6)	3(2.9)	2(5.6)	0.46
Grade I (*n*, %)	8(5.7	4(3.8)	4(11.1)	0.11
Grade II (*n*, %)	12(8.6)	9(8.7)	3(8.3)	0.95
Grade IIIA (*n*, %)	28(20.0)	19(18.3)	9(25.0)	0.38
Grade IIIB (*n*, %)	2(1.4)	2(1.9)	0(0)	0.42
Grade IV (*n*, %)	85(60.7)	67(64.4)	18(50.0)	0.13
Sphenoid sinus pnuematization (*n*, %)	84(60.0)	62(59.6)	22(61.1)	0.88
T2 SI ratio	1.2(1.0–1.4)	1.20(1.00–1.40)	1.26(1.10–1.40)	0.66
Tumor volume (cm^3^)	6.7(3.7–13.1)	6.45(3.85–11.6)	7.10(2.70–16.7)	0.88
Prolactin index (ng/mL∙cm^3^)	443(290–705)	402(288–603)	467(250–745)	0.48
Maximal diameter (mm)	29(24.3–35.4)	28.3(24.2–34.8)	30.1(24.5–46.5)	0.24

Normally distributed continuous variables are described as mean ± SD and median (interquartile range) for non-normally distributed continuous variables, and number (%) for categorical variables. Bold denotes statistical significance at *p* < 0.05.

**Table 2:** Baseline characteristics of bioactive prolactinoma according to hormonal response of cabergoline.

	Total (*n* = 140)	Biochemical Responder (*n* = 112)	Biochemical Non-Responder (*n* = 28)	*p* Value
Demographics				
Age (years)	43 ± 14	44 ± 14	42 ± 17	0.63
Male sex (*n*, %)	108(77.1)	88(78.6)	20(71.4)	0.42
First month CAB dose (mg/week)	2(1–2)	2.0(1.0–2.0)	2.0(1.3–2.8)	0.63
Hormone replacement therapy (*n*, %)	25(17.9)	15(13.4)	10(35.7)	**0.006**
Steroid replacement	10(7.1)	5(4.5)	5(18.5)	**0.011**
Thyroid hormone replacement	16(11.4)	10(8.9)	6(21.4)	0.063
Steroid and thyroid hormone replacement	6(4.3)	3(2.7)	3(10.7)	0.06
Sex hormone replacement	10(7.1)	6(5.4)	4(14.3)	0.10
Hormone deficiency recovery (*n*, %)	7(7.6)	5(4.5)	2(7.1)	0.47
Total CAB dose in 1st year (mg/year)	118(92–144)	100(92–144)	140(92–184)	0.39
Duration of CAB use (months)	65.5(31–90)	68(31–90)	57(21–86)	0.41
**Hormones (Basal)**				
Prolactin (ng/mL)	2592(1584–4775)	2620(1578–4728)	3827(1932–6516)	0.21
FSH (mIU/mL) (Females only)	3.9(2.2–10.9)	6.2(2.0–14.7)	2.8(2.3–2.8)	0.37
Testosterone (ng/dL) (Males only)	124(68.4–202.3)	136(80.1–207.8)	72(30.6–170)	**0.013**
Biochemical & radiological responders (*n*, %)	104(74.3)	104(92.9)	0(0)	<0.001
**Basal radiologic parameters**				
Grade of Knosp				
Grade 0 (*n*, %)	5(3.6)	3(2.7)	2(7.1)	0.26
Grade I (*n*, %)	8(5.7)	4(3.6)	4(14.3)	**0.029**
Grade II (*n*, %)	12(8.6)	10(8.9)	2(7.1)	0.76
Grade IIIA (*n*, %)	28(20.0)	21(18.8)	7(25.0)	0.44
Grade IIIB (*n*, %)	2(1.4)	2(1.8)	0(0)	0.48
Grade IV (*n*, %)	85(60.7)	72(64.3)	13(46.4)	0.089
Sphenoid sinus pnuematization (*n*, %)	84(60.0)	66(58.9)	18(64.3)	0.67
T2 SI ratio	1.2(1.0–1.4)	1.20(1.00–1.40)	1.20(1.03–1.40)	0.97
Tumor volume (cm^3^)	6.7(3.7–13.1)	6.20(3.73–11.5)	7.40(3.20–22.8)	0.30
Prolactin index (ng/mL cm^3^)	443(290–705)	402(290–598)	516(242–752)	0.42
Maximal diameter (mm)	29.0(24.3–35.4)	28.3(24.2–34.3)	31.0(25.5–50.5)	0.075

Normally distributed continuous variables are described as mean ± SD and median (interquartile range) for non-normally distributed continuous variables, and number (%) for categorical variables. Bold denotes statistical significance at *p* < 0.05.

**Table 3:** Clinical characteristics of patients with CSF rhinorrhea compared to hormonal and radiologic responders by propensity score matching.

	CSF Rhinorrhea (*n* = 7)	Responders (*n* = 14)	*p* Value
Demographics			
Age (years)	39.9 ± 8.9	35.9 ± 15.7	0.22
Male sex	6(85.7)	14(100)	0.33
First month CAB dose (mg/week)	2.0(1.0–3.0)	2.0(1.4–2.0)	0.65
Total CAB dose in 1st 3 months (mg)	20(12–32)	40(20–42)	**0.038**
Hormone replacement therapy (*n*, %)	4(57.1)	0(0)	**0.006**
Steroid replacement	3(42.9)	0(0)	**0.026**
Thyroid hormone replacement	3(42.9)	0(0)	**0.026**
Steroid and thyroid hormone replacement	3(42.9)	0(0)	**0.026**
Sex hormone replacement	2(28.6)	0(0)	**0.035**
Hormone deficiency recovery (*n*, %)	1(14.3)	0(0)	NA
Total CAB dose in 1st year (mg/year)	96(48–144)	184(92–186)	**0.024**
Duration of CAB use (months)	49(25–94)	41(21–60)	0.33
**Hormones (Basal)**			
Prolactin (ng/mL)	6128(4042–10200)	5200(3754–7711)	0.65
Testosterone (ng/dL)	115(65–175)	131(61–261)	0.37
**Radiologic features (Basal)**			
Grade of Knosp IV (*n*, %)	7(100)	10(71.4)	0.26
Cystic component (*n*, %)	2(28.6)	7(50.0)	0.64
Hemorrhagic component (*n*, %)	1(14.3)	7(50.0)	0.17
Cavernous sinus involvement (*n*, %)	7(100)	12(85.7)	0.53
Skull base involvement (*n*, %)	7(100)	14(100)	NA
Sphenoid sinus post sellar involvement (*n*, %)	7(100)	10(71.4)	0.26
Sphenoid sinus pneumatization (*n*, %)	7(100)	10(71.4)	0.26
T2 SI ratio	1.2(1.0–1.3)	1.3(1.0–1.6)	0.35
Tumor volume (cm^3^)	25.6(18.4–27.2)	16.9(8.8–24.4)	0.14
Prolactin index (ng/mL∙cm^3^)	283(230–363)	328(158–537)	0.46
Maximal diameter (mm)	43.0(38.0–45.8)	37.2(32.8–43.6)	0.25

Normally distributed continuous variables are described as mean ± SD and median (interquartile range) for non-normally distributed continuous variables, and number (%) for categorical variables. NA; Not available. Bold denotes statistical significance at *p* < 0.05.

**Table 4:** Univariate logistic analysis for incidence of hormonal and radiologic non-responders in bioactive prolactinoma.

	Non-Responders (*n* = 36)	
	aOR(95% CI)	*p*-Value
Demographics		
Age (years)	1.00(0.98–1.03)	0.85
Male sex	1.05(0.42–2.61)	0.92
First month CAB dose (mg/week)	1.25(0.79–1.97)	0.35
Hormone replacement therapy	4.33(1.75–10.74)	**0.002**
Steroid replacement	5.17(1.37–19.58)	**0.016**
Thyroid hormone replacement	3.43(1.18–9.96)	**0.024**
Steroid and thyroid hormone replacement	6.38(1.14–36.44)	**0.037**
Sex hormone replacement	5.00(1.32–18.89)	**0.018**
Hormone deficiency recovery	0.26(0.04–1.69)	0.16
Total CAB dose in 1st year (mg/year)	1.00(0.99–1.01)	0.63
Duration of CAB use (months)	1.00(0.99–1.01)	0.90
**Hormones (Basal)**		
Prolactin (ng/mL)	1.00(1.00–1.00)	0.24
**Radiologic features (Basal)**		
Knosp Grade IV	0.00(0.00–0.00)	0.99
Knosp Grave ≥ 3B	0.51(0.24–1.10)	0.084
Cystic component	1.00(0.47–2.15)	0.99
Hemorrhagic component	1.00(0.43–2.32)	0.99
Cavernous sinus involvement	0.55(0.22–1.37)	0.20
Skull base involvement	0.69(0.06–7.80)	0.76
Sphenoid sinus involvement: Post sellar	1.07(0.49–2.31)	0.88
Sphenoid sinus pneumatization	1.07(0.49–2.31)	0.88
T2 SI ratio	0.94(0.69–1.27)	0.67
Tumor volume (cm^3^)	1.01(0.99–1.04)	0.28
Prolactin index (ng/mL∙cm^3^)	1.00(0.99–1.00)	0.30
Maximal diameter (mm)	1.00(0.99–1.02)	0.60

Bold denotes statistical significance at *p* < 0.05.

**Table 5:** Odds ratios for incidence of hormonal and radiologic non-responders according to hormone deficiency.

	Non-Responders (*n* = 36)	
Model	aOR(95%CI)	*p*-Value
1	4.33(1.75–10.74)	**0.002**
2	5.03(1.96–12.88)	**0.001**
3	4.90(1.90–12.62)	**0.001**
4	5.13(1.96–13.46)	**0.001**

Model 1 no adjustment; Model 2 adjusted for model 1 parameters plus age, male sex, and total cabergoline dose in 1st year (mg/year); Model 3 adjusted for model 2 parameters plus basal prolactin level and basal tumor volume; Model 4 adjusted for model 3 parameters plus sphenoid sinus post sellar involvement and sphenoid sinus pneumatization; bold denotes statistical significance at *p* < 0.05.

## Data Availability

Some or all data sets generated during and/or analyzed during the current study are not publicly available but are available from the corresponding author on reasonable request.

## Supplementary Materials

The following are available online at www.mdpi.com/2072-6694/13/21/5374/s1. Table S1: Baseline characteristics and radiologic characteristics of bioactive prolactinoma according to radiologic response of cabergoline, Table S2: Clinical characteristics of patients with CSF rhinorrhea compared to hormonal and radiologic non-responders by propensity score matching.
