# Supplementary material for: Efficacy and Cerebrospinal Fluid Rhinorrhea after Cabergoline Treatment in Patients with Bioactive Macroprolactinoma
Source: Cancers (Basel). 2021 Oct 26;13(21):5374. doi: 10.3390/cancers13215374 (PMC8582509; doi:10.3390/cancers13215374)
Supplement: Supplementary file 1 [file cancers-13-05374-s001.zip › cancers-1398669-supplementary.pdf]

Supplementary Macterial: Efficacy and Cerebrospinal Fluid Rhinorrhea after Cabergoline Treatment in Patients with Bioactive Macroprolactinoma

Hae-Kyung Kim, Jae-Won Hong, Ju-Hyung Moon, Sung-Soo Ahn, Eui-Hyun Kim, Seung-Koo Lee, Eun-Jig Lee, Yae-Won Park and Cheol-Ryong Ku

Table S1. Baseline characteristics and radiologic characteristics of bioactive prolactinoma according to radiologic response of cabergoline.

	Total (n = 140)	Responder (n = 124)	Non-responder (n = 16)	p-value
**Demographics**				
Age (years)	43 ± 15	43 ± 15	45 ± 14	0.77
Male sex (n, %)	108 (77.1)	96(77.4)	12(75.0)	0.76
First month CAB dose (mg/week)	2 (1-2)	2(1-2)	2(1.3-3)	0.26
Hormone replacement therapy (n, %)	23 (16.4)	19(15.3)	4(25.0)	0.301
Steroid replacement	10 (7.2)	7(5.6)	3(20.0)	0.077
Thyroid hormone replacement	16 (11.4)	13(10.5)	3(18.8)	0.4
Steroid and thyroid hormone replacement	6(4.3)	4(3.2)	2(12.5)	0.085
Sex hormone replacement	10 (7.1)	8(6.5)	2(12.5)	0.32
Hormone deficiency recovery	7(7.6)	6(4.8)	1(6.3)	0.66
Total CAB dose in 1st year (mg/year)	100 (92-144)	136(92-144)	96(81-145)	0.48
Duration of CAB use (months)	66 (31-92)	59(31-89)	84.5(65-100)	0.25
**Hormones (Basal)**				
Prolactin (ng/mL)	2571 (1608-4818)	2676(1610-5350)	2325(1365-4506)	0.43
**Biochemical & radiological responders (n,%)**	104(74.3)	103(83.1)	1(6.3)	**<0.01**
**Radiologic features (Basal)**				
Grade of Knosp				
Grade 0 (n, %)	5 (3.6)	4(3.2)	1(6.3)	0.46
Grade I (n, %)	8 (5.7)	6(4.8)	2(12.5)	0.23
Grade II (n, %)	12 (8.6)	11(8.9)	1(6.3)	0.72
Grade IIIA (n, %)	28 (20.0)	24(19.4)	4(25.0)	0.53
Grade IIIB (n, %)	2 (1.4)	2(1.6)	0(0)	0.61
Grade IV (n, %)	85 (60.7)	77(62.1)	8(50.0)	0.35
Sphenoid sinus pnuematization (n, %)	84 (60.0)	74(59.7)	10(62.5)	0.83
T2 SI ratio	1.4 (1.0-1.4)	1.2(1-1.4)	1.2(0.95-1.4)	0.78
Tumor volume (cm3)	6.5 (6.7-12.1)	6.8(3.9-13.4)	5.6(2.6-11.2)	0.36
Prolactin index (ng/mL · cm^3)	443(290-705)	432(283-663)	351(247-621)	0.77
Maximal diameter (mm)	28.6 (24.1-35.1)	29(24.5-36.0)	28.7(23-34.5)	0.83

Normally distributed continuous variables were described as mean ± SD and median (interquartile range) for non-normally distributed continuous variables, and number (%) for categorical variables. Bold denotes statistical significance at $p < 0.05$

Table S2. Clinical characteristics of patients with CSF rhinorrhea compared to hormonal and radiologic non-responders by propensity score matching.

	CSF rhinorrhea (*n* = 7)	Non-responders (*n* = 14)	*p*-value
**Demographics**			
Age (years)	39.9± 8.9	36.7± 15.3	0.58
Male sex	6(85.7)	12(85.7)	0.99
First month CAB dose (mg/week)	2(1-3)	2(2-3)	0.50
Hormone replacement therapy (n, %)	4(57.1)	4(28.6)	0.35
Steroid replacement (n, %)	3(42.9)	0(0)	**0.03**
Thyroid hormone replacement (n, %)	3(42.9)	2(14.3)	0.28
Steroid and thyroid hormone replacement (n,%)	3(42.9)	0(0)	**0.026**
Sex hormone replacement (n, %)	2(28.6)	3(21.4)	0.99
Hormone deficiency recovery (n,%)	1(14.3)	0(0)	0.99
Total CAB dose (mg/year)	74(48-140)	142(96-157)	0.06
Duration of CAB use (months)	49(25-94)	80(52-95)	0.43
**Hormones (Basal)**			
Prolactin (ng/mL)	6128(4042-10200)	4385(2439-7484)	0.23
**Radiologic features (Basal)**			
Grade of Knosp IV (n, %)	7(100)	8(57.1)	0.061
Cystic component (n, %)	2(28.6)	9(64.3)	0.18
Hemorrhagic component (n, %)	1(14.3)	3(21.4)	0.99
Cavernous sinus involvement (n, %)	7(100)	10(71.4)	0.26
Skull base involvement (n, %)	7(100)	14(100)	NA
Sphenoid sinus post sellar involvement (n,%)	7(100)	9(64.3)	0.12
Sphenoid sinus pneumatization (n, %)	7(100)	9(64.3)	0.12
T2 SI ratio	1.2(1.0-1.3)	1.4(1.2-1.6)	0.07
Tumor volume (cm3)	25.6(18.4-27.2)	12.1(7.9-25.5)	0.07
Prolactin index (ng/mL · cm^3)	283(230-363)	335(166-616)	0.55
Maximal diameter (mm)	43(38-45.8)	35.6(28.8-53.8)	0.85

Normally distributed continuous variables were described as mean ± SD and median (interquartile range) for non-normally distributed continuous variables, and number (%) for categorical variables. Bold denotes statistical significance at $P < 0.05$.
